# The association of reduced lung function with blood pressure variability in African Americans: data from the Jackson Heart Study

**DOI:** 10.1186/s12872-015-0182-2

**Published:** 2016-01-12

**Authors:** John N. Booth III, Nicole Redmond, Mario Sims, Daichi Shimbo, Paul Muntner

**Affiliations:** University of Alabama at Birmingham, Birmingham, AL USA; University of Mississippi Medical Center, Jackson, MS USA; Columbia University Medical Center, New York, NY USA; Department of Epidemiology, University of Alabama at Birmingham, 1700 University Boulevard, LHL 440, Birmingham, AL 35294 USA

**Keywords:** Ambulatory blood pressure, African American, Lung function, FEV1, FVC, Blood pressure variability

## Abstract

**Background:**

African Americans (AAs) have lower lung function, higher blood pressure variability (BPV) and increased risk for hypertension and cardiovascular disease (CVD) compared with whites. The mechanism through which reduced lung-function is associated with increased CVD risk is unclear.

**Methods:**

We evaluated the association between percent predicted lung-function and 24-hour BPV in 1008 AAs enrolled in the Jackson Heart Study who underwent ambulatory blood pressure (BP) monitoring. Lung-function was assessed as forced expiratory volume in one second (FEV1), forced vital capacity (FVC) and the ratio of FEV1-to-FVC during a pulmonary function test using a dry rolling sealed spirometer and grouped into gender-specific quartiles. The pairwise associations of these three lung-function measures with two measures of 24-hour BPV, (1) day-night standard deviation (SD_dn_) and (2) average real variability (ARV) were examined for systolic BP (SBP) and, separately, diastolic BP (DBP).

**Results:**

SD_dn_ of SBP was not associated with FEV1 (mean ± standard deviation from lowest-to-highest quartile: 9.5 ± 2.5, 9.4 ± 2.4, 9.1 ± 2.3, 9.3 ± 2.6; p-trend = 0.111). After age and sex adjustment, the difference in SD_dn_ of SBP was 0.0 (95 % CI −0.4,0.4), −0.4 (95 % CI −0.8,0.1) and −0.3 (95 % CI −0.7,0.1) in the three progressively higher versus lowest quartiles of FEV1 (p-trend = 0.041). Differences in SD_dn_ of SBP across FEV1 quartiles were not statistically significant after further multivariable adjustment. After multivariable adjustment, no association was present between FEV1 and ARV of SBP or SD_dn_ and ARV of DBP or when evaluating the association of FVC and FEV1-to-FVC with 24-hour BPV.

**Conclusion:**

Lung-function was not associated with increased 24-hour BPV.

**Electronic supplementary material:**

The online version of this article (doi:10.1186/s12872-015-0182-2) contains supplementary material, which is available to authorized users.

## Background

Over 40 million US adults have reduced lung function, defined by low forced vital capacity (FVC) or forced expiratory volume in 1 s (FEV1) [1]. Lower lung function has been associated with higher risk for all-cause mortality in African Americans (AAs) compared with whites [2–4]. Low FVC and FEV1 have also been associated with an increased risk for cardiovascular disease (CVD) incidence in several studies that include African Americans [1, 5–8]. However, there are few reports on lung function and CVD mortality in AAs versus whites, but AAs have a higher burden of CVD risk factors than whites [2–4]. The mechanisms by which reduced lung function increases the risk for CVD are unclear.

Within individuals, blood pressure (BP) fluctuates from beat-to-beat, throughout the course of the day and over longer periods of time [9–11]. Similar to reduced lung function, high BP variability (BPV) has been associated with increased CVD risk [11, 12]. Reduced lung function is associated with several correlates of high BPV including older age, higher mean systolic BP (SBP) and inflammation [13–15]. Studies have suggested that an association exists between reduced lung function and increased BPV [10, 11]. However, prior studies have assessed BPV using beat-to-beat measurements during a 5-minute recording and short-term BPV may be directly influenced by breathing mechanics. In contrast, BP measured repeatedly over a longer time period, such as with ambulatory blood pressure monitoring (ABPM), may be more useful in evaluating the association between lung function and BPV.

AAs have lower lung function and higher BPV compared to whites, but few studies have conducted ABPM in AAs [16, 17]. The Jackson Heart Study (JHS), an exclusively AA cohort, assessed BPV over a 24-h period using ABPM in > 1000 participants. The aim of the current study was to evaluate the association between lung function, assessed by percent predicted FEV1 and FVC, with BPV assessed by 24-hour ABPM in adult AAs [18]. The association between the percent predicted FEV1-to-FVC ratio, a measure of airway obstruction, and BPV was also evaluated.

## Methods

### Study population

The design and conduct of the JHS has been described previously [19–21]. Briefly, JHS is a prospective, community-based observational study designed to evaluate CVD risk among AAs. JHS enrolled 5301 non-institutionalized AA participants, aged ≥ 21 years, between 2000 and 2004 from the Atherosclerosis Risk in the Community (ARIC) site in Jackson, Mississippi (30 %) and a regionally representative sample of urban and rural residents from the Jackson, Mississippi metropolitan tri-county region (Hinds, Madison and Rankin counties) that were randomly contacted (17 %), volunteers (22 %), or secondary family members (31 %) [22]. The current analysis was restricted to JHS participants who completed 24-hour ABPM at Exam 1 (*n* = 1148). After excluding participants who did not meet the International Database on Ambulatory blood pressure monitoring in relation to Cardiovascular Outcomes (IDACO) criteria [23] for complete ABPM (described below) and those who did not have valid lung function measures, the analytic sample included 1008 participants. The JHS protocol was approved by the Institutional Review Board governing human subjects research. Informed consent was provided by all participants. The current analysis was approved by the University of Alabama at Birmingham Institutional Review Board.

### Data collection

The current analysis included data collected during an in-home study visit, a clinic examination and 24-hour ABPM period. During the in-home visit, trained study staff completed questionnaires with each participant. These questionnaires were used to collect information on age, sex, physical activity, smoking status and prior diagnosed co-morbid conditions. During the clinic examination, trained technicians measured height, weight and BP, collected blood samples and conducted a pulmonary function test. In addition, based on self-report and a pill bottle review, prescription and over the counter medications taken in the 2 weeks prior to the study visit were recorded. Following the clinic examination, participants were given the opportunity to complete ABPM.

Current smoking was defined by “yes” responses to the two questions “Have you smoked more than 400 cigarettes in your lifetime?” and “Do you now smoke cigarettes?” Participants who reported smoking more than 400 cigarettes but quit smoking ≥ 1 year were categorized as former smokers. Participants who reported not having smoked more than 400 cigarettes in their life were considered never smokers. Pack-years of cigarette smoking were estimated by multiplying the self-reported number of packs of cigarettes smoked daily by the self-reported number of years the person smoked. Using a modified Baecke questionnaire, duration, frequency and intensity of physical activity in four domains (active living, work, home life and sport) were evaluated on a scale of 1 to 5 and summed to calculate a total physical activity score [24]. Higher scores represent more physical activity. History of myocardial infarction (MI), coronary revascularization procedures and stroke were assessed by self-report. Using height and weight measured during the clinic examination, body mass index (BMI) was calculated as weight in kilograms divided by height in meters squared and categorized as non-obese (BMI < 30 kg/m^2^) or obese (BMI ≥ 30 kg/m^2^). Clinic BP was measured twice, separated by 1 min, with an appropriate cuff size using a Hawksley random zero sphygmomanometer (Hawksley and Sons Ltd) after participants had rested for ≥ 5 min. The average of these measurements was recorded. Clinic BP control was defined as a mean clinic SBP < 140 mm Hg and DBP < 90 mm Hg. Total and high-density lipoprotein (HDL) cholesterol was quantified by an oxidase method. High-sensitivity C-reactive protein (CRP) was calculated using the latex particle immunoturbidimetric assay method. Urinary albumin and creatinine were quantified from a 24-hour urine collection using the nephalometric immunoassay and enzymatic methods, respectively. Spot urine albumin and creatinine were used for participants for whom 24-hour urinary albumin or creatinine was not collected. Albuminuria was defined as urinary albumin to urinary creatinine ratio ≥ 30 mg/g. Estimated glomerular filtration rate (eGFR) was calculated using the Chronic Kidney Disease Epidemiology Collaboration (CKD-EPI) equation [25]. Reduced eGFR was defined as a level < 60 ml/min/1.73 m^2^. Diabetes was defined as a fasting (≥8 h) serum glucose ≥ 126 mg/dL or hemoglobin A1c ≥ 6.5 % or use of insulin or oral hypoglycemic medications within 2 weeks prior to the clinic examination visit.

#### Lung function measurements

Three measures of lung function were evaluated: forced expiratory volume in 1 s (FEV1), forced vital capacity (FVC) and the ratio of FEV1-to-FVC. FEV1 is the volume of exhaled gas in 1 s from the beginning of a forced exhalation. FVC is the total volume of exhaled gas from the lung. The ratio of these measures, FEV1 divided by FVC, is used as a marker of airway obstructions. These were measured in accordance with the 1994 American Thoracic Society recommendations by a trained technician during a pulmonary function test using a dry rolling sealed spirometer. The test was performed until automated quality checking software (Occupational Marketing, Inc., Houston, TX) indicated the American Thoracic Society acceptability and reproducibility standards were met or for a maximum of eight attempts. In addition, each spirometry tracing was visually reviewed and scored for quality on three distinct aspects: 1) first volume, 2) second volume and 3) initial effort. Each measure of lung function was normalized for participant’s age, sex and height resulting in percent predicted FEV1, FVC and FEV1-to-FVC, respectively [18].

#### Ambulatory blood pressure monitoring

An ABPM device (Spacelabs 90207) was fitted on each participant’s non-dominant arm using an appropriate cuff size as determined by measuring the arm circumference at the midpoint of the extended upper arm. The ABPM device was programed to record BP every 20 min for 24-hours. Upon returning the device, the data were downloaded, automatically evaluated for quality control and processed using Medifacts International’s Medicom software (Rockville, MD) customized for the JHS. A valid ABPM measurement, defined using IDACO criteria, required participants to have ten or more valid daytime (10:00 to 20:00) and five or more valid nighttime (00:00 to 06:00) SBP and DBP measurements [23]. Two measures of BPV were calculated for the current study, day-night standard deviation (SD_dn_) and average real variability (ARV). SD_dn_ of SBP was calculated as the weighted standard deviation of SBP measured during the daytime and nighttime [26]. ARV of SBP was calculated as the average absolute difference between consecutive SBP readings from the ABPM [12]. SD_dn_ of DBP and ARV of DBP were also calculated.

### Statistical analysis

The distribution of lung function measures differed for men and women. Therefore, sex specific quartiles of FEV1, FVC, and FEV1-to-FVC ratio were created. Quartiles were chosen instead of specific thresholds (e.g., FEV1-to-FVC ratio < 0.70) as few participants met the threshold criteria. Additionally, an association between lower lung function in the normal range and higher blood pressure variability would provide stronger evidence of a possible mechanistic relationship. Participant characteristics were calculated by quartile of FEV1, FVC and FEV1-to-FVC ratio, separately. We analyzed the pairwise associations of three exposures (FEV1, FVC and the FEV1-to-FVC ratio) with two outcomes (SD_dn_ and ARV) for SBP and DBP, separately. Below we describe the analyses for the association between FEV1 and SD_dn_ of SBP. Identical analyses were conducted for FVC and FEV1-to-FVC ratio and for the outcomes of ARV of SBP and SD_dn_ and ARV of DBP.

Mean SD_dn_ of SBP was calculated by quartile of FEV1. Next, the differences in SD_dn_ of SBP for each of the three highest quartiles of FEV1, compared to the lowest quartile (the reference category), were calculated using linear regression. Two models were estimated. The initial model included adjustment for age and sex. A subsequent model had additional adjustment for pack years of cigarette smoking, physical activity, BMI, diabetes, total and HDL-cholesterol, statin use, history of stroke and myocardial infarction, eGFR, albuminuria, C-reactive protein, mean daytime SBP and the classes of antihypertensive medications being taken. Since lung function and BPV have each been associated with sex, smoking status, antihypertensive medication use and BP control, analyses were performed to assess the association between FEV1, FVC and FEV1-to-FVC ratio and SD_dn_ in subgroups defined by sex (male/female), smoking status (never, former, current), antihypertensive medication use (yes/no) and controlled clinic BP (yes/no). Two-sided p-values < 0.05 were considered statistically significant. All analyses were conducted using SAS version 9.3 (SAS Institute, Research Triangle Park, NC).

## Results

### Participant characteristics

Participants in the lowest quartile of FEV1 had higher mean height, weight and BMI, lower physical activity levels and a higher proportion was current smokers (Table 1) compared to the highest quartile. Also, the prevalence of diabetes, a history of stroke and myocardial infarction, hypertension, reduced eGFR, albuminuria and statin use was higher in the lowest versus highest quartile of FEV1. Mean daytime SBP was higher and antihypertensive medication use was more common for participants in the lowest versus highest quartile of FEV1. Similar associations were present between participant characteristics and quartiles of FVC (Table 1). Additional file 1: Table S1 reports participant characteristics by quartile of FEV1-to-FVC ratio.

**Table 1:** Baseline characteristics for Jackson Heart Study participants by quartiles of forced-expiratory-volume-in-1-second and forced-vital-capacity

	Quartile 1 (lowest)	Quartile 2	Quartile 3	Quartile 4 (highest)
	Forced expiratory volume in 1 s			
	(*n* = 251)	(*n* = 253)	(*n* = 253)	(*n* = 251)
FEV1 range in Men, percent predicted	<80.7	80.7 to 91.8	91.8 to 101.1	≥101.1
FEV1 range in Women, percent predicted	<83.8	83.8 to 95.4	95.4 to 106.8	≥106.8
Age, years	59.8 ± 10.4	57.7 ± 10.8	57.9 ± 11.0	61.0 ± 11.1
Male, %	31.9	32.0	32.0	31.9
Height, cm	170.3 ± 9.4	168.6 ± 9.4	167.7 ± 8.6	166.1 ± 8.7
Weight, kg	92.3 ± 19.1	90.2 ± 20.5	85.3 ± 19.3	84.3 ± 19.3
BMI, kg/m^2^	31.8 ± 6.1	31.8 ± 7.3	30.3 ± 6.2	30.5 ± 5.5
Obese, %	60.4	51.0	45.1	43.8
Physical activity score^a^, exercise units	8.2 ± 2.6	8.2 ± 2.3	8.4 ± 2.6	8.5 ± 2.6
Cigarette pack years	9.4 ± 18.3	8.8 ± 17.1	4.5 ± 10.8	6.3 ± 13.8
Smoking status, %				
Never	63.8	64.4	73.9	68.9
Former	21.5	17.4	19.4	24.3
Current	13.2	15.4	5.9	5.2
Diabetes, %	31.1	27.1	19.9	19.3
Total cholesterol, mg/dL	199.6 ± 37.1	201.1 ± 41.2	199.9 ± 37.4	203.6 ± 44.1
High-density lipoprotein, mg/dL	53.4 ± 15.8	54.0 ± 14.5	52.8 ± 14.9	55.2 ± 15.0
Statin use, %	14.7	14.7	13.6	13.1
History of stroke, %	5.2	4.4	1.2	4.4
History of myocardial infarction, %	10.0	7.1	5.9	6.4
eGFR < 60 ml/min/m^2^, %	12.6	6.4	7.2	8.8
ACR ≥ 30, %	9.2	9.5	5.1	8.8
High sensitivity c-reactive protein, mg/L	3.5 (1.5 – 6.6)	3.0 (1.4 – 5.7)	2.8 (1.1 – 5.6)	2.2 (0.9 – 4.6)
Mean daytime systolic blood pressure, mm Hg	131.0 ± 14.8	129.7 ± 13.4	128.5 ± 12.7	128.4 ± 13.0
Mean daytime diastolic blood pressure, mm Hg	77.0 ± 9.4	78.2 ± 9.4	78.4 ± 9.2	77.6 ± 9.0
Hypertension, %	72.1	64.8	66.0	61.8
Antihypertensive medication use^b^, %	67.7	58.5	55.7	55.4
Antihypertensive medication classes^b^, %				
Diuretic	72.6	65.7	62.4	70.5
Beta blocker	28.7	24.3	23.3	17.4
Calcium channel blocker	39.5	37.9	33.1	35.6
Angiotensin converting enzyme inhibitors	44.0	39.3	33.1	39.4
Angiotensin receptor blockers	15.3	8.6	14.3	10.6
	Forced vital capacity			
	(*n* = 251)	(*n* = 253)	(*n* = 253)	(*n* = 251)
FVC range in men, percent predicted	<81.4	81.4 to 90.4	90.4 to 99.8	≥99.8
FVC range in Women, percent predicted	<83.3	83.3 to 94.2	94.2 to 105.2	≥105.2
Age, years	59.5 ± 10.2	57.3 ± 10.5	58.4 ± 11.1	61.1 ± 11.5
Male, %	31.9	32.0	32.0	31.9
Height, cm	170.1 ± 9.8	168.2 ± 8.8	167.6 ± 8.5	166.8 ± 9.1
Weight, kg	93.9 ± 19.7	89.4 ± 20.9	85.6 ± 16.9	83.2 ± 17.6
BMI, kg/m^2^	32.4 ± 6.2	31.6 ± 7.1	30.5 ± 5.9	29.9 ± 5.9
Obese, %	63.2	53.4	45.5	38.3
Physical activity score^a^, exercise units	8.1 ± 2.5	8.3 ± 2.4	8.3 ± 2.6	8.6 ± 2.7
Cigarette pack years	7.9 ± 15.6	7.9 ± 16.4	6.0 ± 13.8	7.1 ± 15.5
Smoking status, %				
Never	67.7	64.0	71.9	67.3
Former	17.5	20.6	19.8	24.7
Current	12.4	13.8	7.1	6.4
Diabetes, %	32.8	23.0	23.5	18.1
Total cholesterol, mg/dL	201.1 ± 40.9	200.6 ± 37.9	20.6 ± 42.0	199.0 ± 39.5
High-density lipoprotein, mg/dL	53.7 ± 15.3	53.1 ± 14.6	53.4 ± 15.3	55.2 ± 15.1
Statin use, %	17.3	10.0	15.2	13.6
History of stroke, %	5.2	4.4	2.4	3.2
History of myocardial infarction, %	10.0	5.9	5.1	8.4
eGFR < 60 ml/min/m^2^, %	11.4	7.5	4.8	11.3
ACR ≥ 30, %	12.8	6.3	6.3	7.2
High sensitivity c-reactive protein, mg/L	3.4 (1.7 – 6.6)	3.1 (1.2 – 5.7)	2.8 (1.2 – 5.7)	1.9 (0.9 – 4.1)
Mean daytime systolic blood pressure, mm Hg	131.4 ± 14.5	129.2 ± 12.9	129.5 ± 13.1	128.6 ± 13.3
Mean daytime diastolic blood pressure, mm Hg	76.9 ± 9.3	78.8 ± 9.4	77.8 ± 8.7	77.6 ± 9.4
Prevalent hypertension, %	72.5	66.0	65.2	61.0
Antihypertensive medication use^b^, %	67.7	58.1	56.5	55.0
Antihypertensive medications^b^, %				
Diuretic	74.1	61.2	64.5	71.7
Beta blocker	23.4	28.8	23.9	18.1
Calcium channel blocker	41.1	35.3	37.0	32.3
Angiotensin converting enzyme inhibitors	45.6	33.8	37.0	39.4
Angiotensin receptor blockers	12.7	11.5	15.2	9.5

Numbers in table are percentages or mean ± standard deviation except high-sensitivity c-reactive protein which is median (25th – 75th percentiles)

^a^Higher score = more physical activity

eGFR: estimated glomerular filtration rate; ACR: albumin to creatinine ratio

^b^Among participants taking ≥ 1 antihypertensive medication

### FEV1 and FVC with day-night standard deviation and average real variability of BP

There were no graded associations of lower quartiles of FEV1 and, separately, FVC with higher mean SD_dn_ of SBP (Table 2). An association between FEV1 and SD_dn_ of SBP was present after adjustment for age and sex but was attenuated after multivariable adjustment. The association between lower FVC with higher SD_dn_ of SBP after adjustment for age and sex was attenuated after full multivariable adjustment. FEV1 and FVC were not associated with SD_dn_ of DBP before or after multivariable adjustment. Associations were not present between FEV1 and FVC with ARV of SBP and DBP (Table 3).

**Table 2:** Differences in day-night standard deviation of blood pressure across quartiles of forced-expiratory-volume-in-1-second and forced-vital-capacity

	Quartile 1 (lowest)	Quartile 2	Quartile 3	Quartile 4 (highest)	p-trend
	Forced expiratory volume in 1 s				
Systolic blood pressure	(*n* = 251)	(*n* = 253)	(*n* = 253)	(*n* = 251)	
Mean ± standard deviation	9.5 ± 2.5	9.4 ± 2.4	9.1 ± 2.3	9.3 ± 2.6	0.111
Model 1, β (95 % CI)	0 (ref)	0.0 (−0.4 to 0.4)	−0.4 (−0.8 to 0.1)	−0.3 (−0.7 to 0.1)	0.041
Model 2, β (95 % CI)	0 (ref)	0.1 (−0.4 to 0.5)	−0.1 (−0.5 to 0.3)	−0.0 (−0.4 to 0.4)	0.775
Diastolic blood pressure					
Mean ± standard deviation	8.1 ± 2.1	8.2 ± 2.1	8.1 ± 2.3	8.0 ± 2.0	0.318
Model 1, β (95 % CI)	0 (ref)	0.0 (−0.4 to 0.4)	−0.1 (−0.4 to 0.3)	−0.2 (−0.6 to 0.2)	0.321
Model 2, β (95 % CI)	0 (ref)	−0.1 (−0.5 to 0.3)	−0.1 (−0.5 to 0.3)	−0.2 (−0.6 to 0.2)	0.393
	Forced vital capacity				
Systolic blood pressure	(*n* = 251)	(*n* = 253)	(*n* = 253)	(*n* = 251)	
Mean ± standard deviation	9.6 ± 2.5	9.3 ± 2.5	9.1 ± 2.4	9.3 ± 2.4	0.054
Model 1, β (95 % CI)	0 (ref)	−0.2 (−0.6 to 0.2)	−0.5 (−0.9 to −0.1)	−0.5 (−0.9 to −0.1)	0.009
Model 2, β (95 % CI)	0 (ref)	−0.1 (−0.6 to 0.3)	−0.3 (−0.7 to 0.2)	−0.1 (−0.5 to 0.3)	0.495
Diastolic blood pressure					
Mean ± standard deviation	8.3 ± 2.1	8.1 ± 2.3	7.8 ± 2.1	8.1 ± 2.1	0.155
Model 1, β (95 % CI)	0 (ref)	−0.1 (−0.5 to 0.2)	−0.5 (−0.8 to −0.1)	−0.2 (−0.6 to 0.2)	0.157
Model 2, β (95 % CI)	0 (ref)	−0.3 (−0.7 to 0.1)	−0.5 (−0.9 to −0.1)	−0.1 (−0.5 to 0.3)	0.348

Forced expiratory volume in 1 s quartile cut points (lowest to highest quartile):

Men: < 80.7, 80.7 to 91.8, 91.8 to 101.1, and ≥ 101.1

Women: < 83.8, 83.8 to 95.4, 95.4 to 106.8, and ≥ 106.8

Forced vital capacity quartile cut points (lowest to highest quartile):

Men: < 81.4, 81.4 to 90.4, 90.4 to 99.8, and ≥ 99.8

Women: < 83.3, 83.3 to 94.2, 94.2 to 105.2, and ≥ 105.2

CI: confidence interval

Model 1: adjustment for demographics (age and sex)

Model 2: Adjustment for Model 1 variables plus behaviors (pack years of cigarette smoking, physical activity, and body mass index), co-morbid conditions (diabetes, total and HDL-cholesterol and statin use, history of stroke and history of myocardial infarction), kidney function (estimated glomerular filtration rate and albuminuria), markers of inflammation (C-reactive protein), mean daytime SBP or DBP and antihypertensive medication classes being taken

**Table 3:** Differences in average real variability of blood pressure across quartiles of forced-expiratory-volume-in-1-second and forced-vital-capacity

	Quartile 1 (lowest)	Quartile 2	Quartile 3	Quartile 4 (highest)	p-trend
	Forced expiratory volume in 1 s				
Systolic blood pressure	(*n* = 251)	(*n* = 253)	(*n* = 253)	(*n* = 251)	
Mean ± standard deviation	8.8 ± 2.0	8.6 ± 2.0	8.7 ± 2.1	8.7 ± 2.2	0.593
Model 1, β (95 % CI)	0 (ref)	−0.1 (−0.4 to 0.3)	0.0 (−0.3 to 0.4)	−0.2 (−0.6 to 0.1)	0.349
Model 2, β (95 % CI)	0 (ref)	0.0 (−0.3 to 0.4)	0.2 (−0.2 to 0.5)	0.0 (−0.3 to 0.4)	0.627
Diastolic blood pressure					
Mean ± standard deviation	7.7 ± 2.0	7.6 ± 2.0	7.7 ± 2.3	7.6 ± 2.1	0.635
Model 1, β (95 % CI)	0 (ref)	−0.1 (−0.5 to 0.2)	−0.0 (−0.4 to 0.3)	−0.1 (−0.5 to 0.2)	0.646
Model 2, β (95 % CI)	0 (ref)	−0.2 (−0.6 to 0.2)	−0.0 (−0.4 to 0.4)	−0.1 (−0.4 to 0.3)	0.993
	Forced vital capacity				
Systolic blood pressure	(*n* = 251)	(*n* = 253)	(*n* = 253)	(*n* = 251)	
Mean ± standard deviation	8.9 ± 1.9	8.6 ± 2.2	8.6 ± 2.0	8.8 ± 2.1	0.617
Model 1, β (95 % CI)	0 (ref)	−0.1 (−0.5 to 0.2)	−0.3 (−0.6 to 0.1)	−0.2 (−0.5 to 0.2)	0.263
Model 2, β (95 % CI)	0 (ref)	−0.1 (−0.4 to 0.3)	−0.1 (−0.5 to 0.2)	0.1 (−0.3 to 0.4)	0.856
Diastolic blood pressure					
Mean ± standard deviation	7.9 ± 2.0	7.6 ± 2.2	7.5 ± 2.1	7.7 ± 2.2	0.212
Model 1, β (95 % CI)	0 (ref)	−0.3 (−0.6 to 0.1)	−0.4 (−0.8 to −0.0)	−0.2 (−0.6 to 0.2)	0.221
Model 2, β (95 % CI)	0 (ref)	−0.3 (−0.7 to 0.0)	−0.4 (−0.8 to −0.0)	−0.1 (−0.4 to 0.3)	0.742

Forced expiratory volume in 1 s quartile cut points (lowest to highest quartile):

Men: < 80.7, 80.7 to 91.8, 91.8 to 101.1, and ≥ 101.1

Women: < 83.8, 83.8 to 95.4, 95.4 to 106.8, and ≥ 106.8

Forced vital capacity quartile cut points (lowest to highest quartile):

Men: < 81.4, 81.4 to 90.4, 90.4 to 99.8, and ≥ 99.8

Women: < 83.3, 83.3 to 94.2, 94.2 to 105.2, and ≥ 105.2

CI: confidence interval

Model 1: adjustment for demographics (age and sex)

Model 2: Adjustment for Model 1 variables plus behaviors (pack years of cigarette smoking, physical activity, and body mass index), co-morbid conditions (diabetes, total and HDL-cholesterol and statin use, history of stroke and history of myocardial infarction), kidney function (estimated glomerular filtration rate and albuminuria), markers of inflammation (C-reactive protein), mean daytime SBP or DBP and antihypertensive medication classes being taken

### FEV1-to-FVC ratio with day-night standard deviation and average real variability of BP

A graded association between lower quartiles of FEV1-to-FVC ratio with higher mean SD_dn_ and ARV of SBP and DBP was not present (Table 4). FEV1-to-FVC ratio was not associated with SD_dn_ or ARV of SBP and DBP before or after multivariable adjustment.

**Table 4:** Differences in blood pressure for measures of blood pressure variability across quartiles of forced-expiratory-volume-in-1-second-to-forced-vital-capacity ratio

	Forced expiratory volume in 1 s to forced vital capacity ratio				
	Quartile 1 (lowest)	Quartile 2	Quartile 3	Quartile 4 (highest)	p-trend
	Day-night standard deviation				
Systolic blood pressure	(*n* = 251)	(*n* = 253)	(*n* = 253)	(*n* = 251)	
Mean ± standard deviation	9.3 ± 2.6	9.2 ± 2.2	9.4 ± 2.5	9.3 ± 2.5	0.680
Model 1, β (95 % CI)	0 (ref)	−0.1 (−0.5 to 0.3)	0.2 (−0.2 to 0.6)	−0.1 (−0.5 to 0.4)	0.890
Model 2, β (95 % CI)	0 (ref)	−0.0 (−0.5 to 0.4)	0.1 (−0.4 to 0.5)	−0.1 (−0.5 to 0.4)	0.314
Diastolic blood pressure					
Mean ± standard deviation	8.1 ± 2.2	8.1 ± 2.0	8.2 ± 2.3	8.0 ± 2.1	0.685
Model 1, β (95 % CI)	0 (ref)	0.0 (−0.4 to 0.4)	0.1 (−0.3 to 0.4)	−0.1 (−0.5 to 0.3)	0.688
Model 2, β (95 % CI)	0 (ref)	−0.0 (−0.4 to 0.3)	−0.2 (−0.6 to 0.2)	−0.2 (−0.6 to 0.2)	0.180
	Average real variability				
Systolic blood pressure					
Mean ± standard deviation	8.7 ± 2.2	8.6 ± 2.0	8.8 ± 2.0	8.8 ± 2.1	0.570
Model 1, β (95 % CI)	0 (ref)	−0.1 (−0.5 to 0.2)	0.1 (−0.2 to 0.5)	−0.0 (−0.4 to 0.3)	0.765
Model 2, β (95 % CI)	0 (ref)	−0.1 (−0.5 to 0.2)	0.1 (−0.3 to 0.4)	0.0 (−0.3 to 0.4)	0.702
Diastolic blood pressure					
Mean ± standard deviation	7.6 ± 2.1	7.7 ± 2.0	7.7 ± 2.2	7.6 ± 2.1	0.888
Model 1, β (95 % CI)	0 (ref)	0.0 (−0.3 to 0.4)	0.1 (−0.2 to 0.5)	0.0 (−0.4 to 0.4)	0.878
Model 2, β (95 % CI)	0 (ref)	−0.1 (−0.4 to 0.3)	−0.1 (−0.5 to 0.3)	−0.2 (−0.6 to 0.2)	0.297

Forced expiratory volume in 1 s: Forced vital capacity ratio quartile cut points (lowest to highest quartile):

Men: < 0.96, 0.96 to 1.02, 1.02 to 1.07, and ≥ 1.07

Women: < 0.97, 0.97 to 1.03, 1.03 to 1.07, and ≥ 1.07

CI: confidence interval

Model 1: adjustment for demographics (age and sex)

Model 2: Adjustment for Model 1 variables plus behaviors (cigarette smoking, physical activity, and body mass index), co-morbid conditions (diabetes, total and HDL-cholesterol and statin use, history of stroke and history of myocardial infarction), kidney function (estimated glomerular filtration rate and albuminuria), markers of inflammation (C-reactive protein), mean daytime SBP or DBP and antihypertensive medication classes being taken

### Sub-group analyses

Within each sub-group investigated, lower quartiles of FEV1 were not associated with higher mean SD_dn_ of SBP and DBP (Additional file 2: Table S2). There were no associations between FVC and quartiles of SD_dn_ of SBP and DBP in each sub-group except for lower quartiles of FVC with higher SD_dn_ of SBP and DBP in participants not taking antihypertensive medications and SD_dn_ of DBP in those without controlled blood pressure (Additional file 3: Tables S3). After multivariable adjustment, there were no associations across quartiles of FEV1 or FVC with SD_dn_ of SBP and DBP in any of the sub-groups. There were no associations across quartiles of FEV1-to-FVC ratio with SD_dn_ of SBP and DBP (Additional file 4: Table S4).

## Discussion

The current analysis of a large, community-based sample of AAs tested the hypothesis that reduced lung function is associated with higher 24-hour BPV. Higher quartiles of FEV1, FVC and FEV1-to-FVC ratio were not associated with higher SD_dn_ and ARV of SBP and DBP in unadjusted analyses and after multivariable adjustment. Additionally, no associations were present between lung function and BPV in sub-groups defined by sex, smoking status, antihypertensive medication use and BP control.

Older age, male sex, AA race, higher 24-hour mean SBP, inflammation, emotion, physical activity, environmental temperature, food, and alcohol and tobacco consumption have been associated with higher BPV [16, 17]. Reduced lung function has been associated with several of these factors [13–15]. These shared associations provided a foundation for the hypothesis of the current study that reduced lung function would be associated with higher BPV. Although there was an association between reduced FEV1 and FVC and higher SD_dn_ of SBP after adjustment for age and sex, it was no longer present after multivariable adjustment.

One way the sympathetic nervous system controls cardiac output, and may subsequently influence BPV, is through lung function. Impaired lung function increases the cardiac oxygen supply-to-demand ratio resulting in higher cardiac output and possibly higher BPV [9]. Two prior studies using beat-to-beat BP measurements have suggested an association between reduced lung function and increased BPV [10, 11]. In both studies, systolic BPV was measured using a continuous 5-minute BP recording. Each of these studies found an association between lower FEV1 and FVC and higher beat-to-beat systolic BPV. However, many factors during a short measurement period that promote an elevated breathing rate response, including increased stress/anxiety, may influence beat-to-beat BPV. The current study estimated BPV over a longer period of time using ABPM and no association was present between reduced lung function and BPV. The results herein suggest that investigations should be undertaken to determine the role of other possible mechanisms, such as neuro-hormonal activation, of higher BPV [27]. For example, hypoxia can increase plasma norepinephrine, norepinephrine myocardial turnover and cardiac oxygen demand [27]. The effect of the sympathetic nervous system activity becoming elevated and poor cardiac function may result in reduced lung function [27]. Compared with breathing mechanics, gas exchange/diffusion, measurable by plethysmography, and other neuro-hormonal activators may be important factors contributing to higher BPV and CVD risk.

BPV derived from ABPM has been associated with an increased risk for CVD and all-cause mortality [12, 26, 28]. For example, in a pooled cohort of 8938 individuals, the hazard ratio (95 % confidence interval) of mortality associated with each standard deviation higher SD_dn_ SBP and DBP was 1.08 (1.01 – 1.16) and 1.16 (1.09 – 1.23), respectively [12]. Similar associations were present for ARV [12]. Despite the results of the current study, BPV still remains an important risk factor for CVD. However, the null association between reduced lung function and higher BPV of the current study suggests that BPV does not mediate the association of lung function with CVD outcomes reported in prior studies [1, 5–8, 29–34]. Future studies should consider investigating whether reducing BPV will lower the risk for target-organ damage and CVD-related outcomes.

Epidemiologic studies have reported an association between reduced lung function and an increased risk of CVD [35]. For example, among 7 058 men and 8353 women in the Renfrew and Paisley study, the hazard ratio (95 % confidence interval) for ischemic heart disease associated with the lowest versus highest quintile of FEV1 expressed as percentage of predictive value (<73 versus >107 for men and <75 and >112 for women) was 1.56 (1.26 – 1.92) and 1.88 (1.44 – 2.47), respectively [7]. Additionally, CVD is the primary cause of death among individuals with reduced lung function [35]. Although an association between lower lung function and incident CVD has been reported in several studies, the underlying mechanism by which reduced lung function predicts CVD outcomes remains unclear. The current results suggest that reduced lung function is not associated with higher 24-hour BPV suggesting other mechanisms underlie the increased CVD risk among individuals with reduced lung function. Future studies should investigate factors that explain the association between reduced lung function and increased CVD risk.

Several limitations should be considered when interpreting the results from the current analysis. Lung function and ABPM have only been measured at a single JHS visit. The cross-sectional study design prevented the assessment of the longitudinal association between lung function and BPV. Additionally, not all JHS participants had ABPM measurements. However, we do not believe our results are due to lack of power because the magnitude of the association between lung function and BPV was too small to be clinically meaningful. Despite these limitations, there are several strengths. The JHS is a large population-based investigation of AAs. Compared to whites, AAs have lower lung function, higher BPV and an increased risk for hypertension and CVD. Few studies that have measured ABP included AAs. Finally, measurements of lung function, BPV and covariates were conducted following a standardized protocol.

## Conclusion

In conclusion, although individuals in the current study with lower lung function had higher 24-hour BPV, no association was present after multivariable adjustment. Future studies are needed to identify the factors underlying higher levels of BPV. Studies are also needed to determine factors that mediate the increased CVD risk associated with reduced lung function.

## Additional files

Additional file 1: Table S1. Baseline characteristics for Jackson Heart Study participants by quartiles of forced-expiratory-volume-in-1-second-to-forced-vital-capacity ratio. (DOCX 24 kb) Additional file 2: Table S2. Difference in day-night standard deviation of blood pressure across quartiles forced-expiratory-volume-in-1-second by subgroups. (DOCX 27 kb) Additional file 3: Table S3. Differences in day-night standard deviation of blood pressure across quartiles of forced-vital-capacity by subgroups. (DOCX 28 kb) Additional file 4: Table S4. Difference in day-night standard deviation of blood pressure across quartiles forced-expiratory-volume-in-1-second-to-forced-vital-capacity by subgroups. (DOCX 28 kb)

## Abbreviations

AA: African American
BPV: Blood pressure variability
BP: Blood pressure
FEV1: Forced expiratory volume in one second
FVC: Forced vital capacity
FEV1-to-FVC: Forced expiratory volume in one second to forced vital capacity
SD_dn_: Day-night standard deviation
ARV: Average real variability
SBP: Systolic blood pressure
DBP: Diastolic blood pressure
JHS: Jackson Heart Study
ARIC: Atherosclerosis Risk in the Community Study
US: United States
CVD: Cardiovascular disease
ABPM: Ambulatory blood pressure monitoring
IDACO: International Database on Ambulatory blood pressure monitoring in relation to Cardiovascular Outcomes
BMI: Body mass index
HDL: High-density lipoprotein cholesterol
CRP: High-sensitivity C-reactive protein
eGFR: Estimated glomerular filtration rate
CKD-EPI: Chronic Kidney Disease Epidemiology Collaboration
CI: Confidence interval
