# Supplementary material for: The association of reduced lung function with blood pressure variability in African Americans: data from the Jackson Heart Study
Source: BMC Cardiovasc Disord. 2016 Jan 12;16:6. doi: 10.1186/s12872-015-0182-2 (PMC4709870; doi:10.1186/s12872-015-0182-2)
Supplement: Additional file 1: Table S1. — Baseline characteristics for Jackson Heart Study participants by quartiles of forced-expiratory-volume-in-1-second-to-forced-vital-capacity ratio. (DOCX 24 kb) [file 12872_2015_182_MOESM1_ESM.docx]

Supplemental Table 1. Baseline characteristics for Jackson Heart Study participants by quartiles of forced-expiratory-volume-in-1-second-to-forced-vital-capacity ratio.

|  | Quartile 1  (lowest) | Quartile 2 | Quartile 3 | Quartile 4  (highest) |
| --- | --- | --- | --- | --- |
|  | **Forced expiratory volume in 1 second to forced vital capacity ratio** | | | |
|  | (n = 251) | (n = 253) | (n = 253) | (n = 251) |
| FEV1-to-FVC ratio range in Men, percent predicted | < 0.96 | 0.96 to 1.02 | 1.02 to 1.07 | ≥ 1.07 |
| FEV1-to-FVC ratio range in Women, percent predicted | < 0.97 | 0.97 to 1.03 | 1.03 to 1.07 | ≥ 1.07 |
| Age, years | 59.1 ± 11.5 | 58.6 ± 11.2 | 58.5 ± 10.7 | 60.1 ± 10.2 |
| Male, % | 31.9 | 32.0 | 32.0 | 31.9 |
| Height, cm | 169.0 ± 9.4 | 168.9 ± 9.0 | 167.6 ± 8.8 | 167.3 ± 9.3 |
| Weight, kg | 85.1 ± 17.5 | 88.6 ± 19.7 | 89.7 ± 21.3 | 88.6 ± 18.1 |
| BMI, kg/m^2^ | 29.9 ± 6.1 | 31.0 ± 6.5 | 31.9 ± 6.7 | 31.6 ± 5.9 |
| Obese, % | 43.2 | 48.6 | 51.8 | 56.6 |
| Physical activity score^a^, exercise units | 8.5 ± 2.6 | 8.5 ± 2.6 | 8.2 ± 2.5 | 8.2 ± 2.6 |
| Cigarette pack years | 9.4 ± 17.5 | 8.0 ± 16.5 | 6.8 ± 15.1 | 4.7 ± 11.3 |
| Smoking status, % |  |  |  |  |
| Never | 61.0 | 67.2 | 69.2 | 73.7 |
| Former | 22.3 | 21.7 | 22.1 | 16.3 |
| Current | 14.7 | 9.9 | 7.1 | 8.0 |
| Diabetes, % | 22.4 | 24.3 | 27.2 | 23.4 |
| Total cholesterol, mg/dL | 196.9 ± 38.1 | 202.2 ± 37.7 | 198.5 ± 37.5 | 206.5 ± 45.7 |
| High-density lipoprotein, mg/dL | 55.1 ± 16.0 | 52.6 ± 15.2 | 52.8 ± 13.3 | 54.9 ± 15.4 |
| Statin use, % | 9.8 | 17.0 | 16.1 | 13.0 |
| History of stroke, % | 2.4 | 4.0 | 4.7 | 4.0 |
| History of myocardial infarction, % | 8.8 | 7.5 | 7.1 | 6.0 |
| eGFR < 60 ml/min/m^2^, % | 9.7 | 6.8 | 8.0 | 10.5 |
| ACR ≥ 30, % | 5.2 | 6.7 | 9.1 | 11.6 |
| High sensitivity c-reactive protein, mg/L | 3.0 (1.2 – 5.7) | 2.5 (1.0 – 4.7) | 3.0 (1.2 – 6.4) | 3.0 (1.3 – 5.7) |
| Mean daytime systolic blood pressure, mm Hg | 129.7 ± 13.9 | 129.2 ± 13.9 | 129.6 ± 13.9 | 129.1 ± 12.3 |
| Mean daytime diastolic blood pressure, mm Hg | 77.3 ± 9.9 | 77.9 ± 8.9 | 78.2 ± 9.2 | 77.8 ± 8.9 |
| Hypertension, % | 66.1 | 63.6 | 65.6 | 69.3 |
| Antihypertensive medication use^b^, % | 60.6 | 56.9 | 58.1 | 61.8 |
| Antihypertensive medication classes^b^, % |  |  |  |  |
| Diuretic | 69.0 | 64.0 | 72.3 | 66.7 |
| Beta blocker | 29.6 | 26.5 | 20.4 | 18.4 |
| Calcium channel blocker | 30.3 | 38.2 | 39.4 | 38.8 |
| Angiotensin converting enzyme inhibitors | 37.3 | 36.8 | 43.8 | 38.8 |
| Angiotensin receptor blockers | 14.1 | 12.5 | 10.2 | 12.2 |

Numbers in table are percentages or mean ± standard deviation except high-sensitivity c-reactive protein which is median (25^th^ – 75^th^ percentiles).

^a^Higher score = more physical activity.

eGFR: estimated glomerular filtration rate; ACR: albumin to creatinine ratio.

^b^Among participants taking ≥ 1 antihypertensive medication.
