# Supplementary material for: The association of reduced lung function with blood pressure variability in African Americans: data from the Jackson Heart Study
Source: BMC Cardiovasc Disord. 2016 Jan 12;16:6. doi: 10.1186/s12872-015-0182-2 (PMC4709870; doi:10.1186/s12872-015-0182-2)
Supplement: Additional file 2: Table S2. — Difference in day-night standard deviation of blood pressure across quartiles forced-expiratory-volume-in-1-second by subgroups. (DOCX 27 kb) [file 12872_2015_182_MOESM2_ESM.docx]

Supplemental Table 2. Difference in day-night standard deviation of blood pressure across quartiles forced-expiratory-volume-in-1-second by subgroups.

|  | **Force expiratory volume in 1 second** | | | |  |
| --- | --- | --- | --- | --- | --- |
|  | Quartile 1  (lowest) | Quartile 2 | Quartile 3 | Quartile 4  (highest) | p-trend |
|  | **Sex** | | | |  |
| **Systolic blood pressure** |  |  |  |  |  |
| **Men** |  |  |  |  |  |
| Mean ± standard deviation | 9.3 ± 2.2 | 9.7 ± 2.4 | 8.8 ± 2.1 | 9.1 ± 2.3 | 0.160 |
| β (95% CI) | 0 (ref) | 0.3 (-0.4 to 1.1) | 0.1 (-0.7 to 0.9) | 0.1 (-0.7 to 0.8) | 0.946 |
| **Women** |  |  |  |  |  |
| Mean ± standard deviation | 9.6 ± 2.7 | 9.2 ± 2.4 | 9.2 ± 2.4 | 9.4 ± 2.7 | 0.314 |
| β (95% CI) | 0 (ref) | -0.0 (-0.6 to 0.5) | -0.2 (-0.7 to 0.3) | -0.0 (-0.6 to 0.5) | 0.711 |
| **Diastolic blood pressure** |  |  |  |  |  |
| **Men** |  |  |  |  |  |
| Mean ± standard deviation | 8.0 ± 2.2 | 8.3 ± 2.1 | 8.1 ± 2.0 | 8.0 ± 1.9 | 0.652 |
| β (95% CI) | 0 (ref) | 0.1 (-0.6 to 0.9) | 0.2 (-0.5 to 1.0) | -0.2 (-0.9 to 0.6) | 0.633 |
| **Women** |  |  |  |  |  |
| Mean ± standard deviation | 8.2 ± 2.1 | 8.1 ± 2.1 | 8.1 ± 2.5 | 8.0 ± 2.1 | 0.371 |
| β (95% CI) | 0 (ref) | -0.2 (-0.6 to 0.3) | -0.3 (-0.8 to 0.2) | -0.2 (-0.7 to 0.2) | 0.281 |
|  | **Smoking status** | | | |  |
| **Systolic blood pressure** |  |  |  |  |  |
| **Never** |  |  |  |  |  |
| Mean ± standard deviation | 9.5 ± 2.6 | 9.3 ± 2.4 | 9.0 ± 2.2 | 9.2 ± 2.8 | 0.244 |
| β (95% CI) | 0 (ref) | 0.1 (-0.4 to 0.7) | -0.1 (-0.7 to 0.4) | -0.1 (-0.6 to 0.5) | 0.632 |
| **Former** |  |  |  |  |  |
| Mean ± standard deviation | 9.4 ± 2.2 | 9.5 ± 2.2 | 9.2 ± 2.6 | 9.2 ± 2.1 | 0.610 |
| β (95% CI) | 0 (ref) | 0.3 (-0.7 to 1.3) | 0.1 (-0.8 to 1.1) | 0.2 (-0.7 to 1.1) | 0.739 |
| **Current** |  |  |  |  |  |
| Mean ± standard deviation | 10.3 ± 2.6 | 9.7 ± 2.5 | 9.5 ± 2.8 | 9.8 ± 1.9 | 0.354 |
| β (95% CI) | 0 (ref) | -0.3 (-1.5 to 0.9) | 0.5 (-1.1 to 2.0) | 2.0 (0.1 to 3.9) | 0.065 |
| **Diastolic blood pressure** |  |  |  |  |  |
| **Never** |  |  |  |  |  |
| Mean ± standard deviation | 8.1 ± 2.1 | 8.0 ± 2.1 | 7.9 ± 1.9 | 7.9 ± 2.1 | 0.431 |
| β (95% CI) | 0 (ref) | -0.1 (-0.6 to 0.4) | -0.2 (-0.6 to 0.3) | -0.2 (-0.7 to 0.3) | 0.490 |
| **Former** |  |  |  |  |  |
| Mean ± standard deviation | 8.0 ± 2.1 | 8.2 ± 2.1 | 8.6 ± 3.4 | 8.0 ± 1.9 | 0.858 |
| β (95% CI) | 0 (ref) | 0.3 (-0.7 to 1.4) | 0.2 (-0.8 to 1.3) | -0.0 (-0.9 to 0.9) | 0.911 |
| **Current** |  |  |  |  |  |
| Mean ± standard deviation | 8.8 ± 2.2 | 8.6 ± 1.9 | 8.5 ± 2.5 | 8.3 ± 2.2 | 0.428 |
| β (95% CI) | 0 (ref) | -0.6 (-1.8 to 0.5) | 0.0 (-1.4 to 1.5) | -0.3 (-2.0 to 1.5) | 0.920 |
|  | **Antihypertensive medication use** | | | |  |
| **Systolic blood pressure** |  |  |  |  |  |
| **Yes** |  |  |  |  |  |
| Mean ± standard deviation | 9.7 ± 2.6 | 9.9 ± 2.5 | 9.3 ± 2.4 | 9.8 ± 2.8 | 0.719 |
| β (95% CI) | 0 (ref) | 0.2 (-0.4 to 0.8) | -0.3 (-0.9 to 0.3) | 0.3 (-0.3 to 0.9) | 0.691 |
| **No** |  |  |  |  |  |
| Mean ± standard deviation | 9.1 ± 2.3 | 8.7 ± 2.1 | 8.7 ± 2.2 | 8.6 ± 2.2 | 0.158 |
| β (95% CI) | 0 (ref) | -0.1 (-0.7 to 0.5) | 0.2 (-0.4 to 0.8) | -0.4 (-1.0 to 0.2) | 0.393 |
| **Diastolic blood pressure** |  |  |  |  |  |
| **Yes** |  |  |  |  |  |
| Mean ± standard deviation | 8.2 ± 2.2 | 8.3 ± 2.4 | 8.0 ± 2.5 | 8.3 ± 2.1 | 0.921 |
| β (95% CI) | 0 (ref) | -0.0 (-0.6 to 0.5) | -0.5 (-1.0 to 0.1) | 0.1 (-0.5 to 0.6) | 0.863 |
| **No** |  |  |  |  |  |
| Mean ± standard deviation | 8.1 ± 1.9 | 8.0 ± 1.7 | 8.2 ± 2.1 | 7.6 ± 1.9 | 0.092 |
| β (95% CI) | 0 (ref) | -0.2 (-0.8 to 0.4) | 0.3 (-0.3 to 0.8) | -0.5 (-1.1 to 0.0) | 0.203 |
|  | **Controlled blood pressure status** | | | |  |
| **Systolic blood pressure** |  |  |  |  |  |
| **Yes** |  |  |  |  |  |
| Mean ± standard deviation | 9.2 ± 2.4 | 9.0 ± 2.1 | 9.0 ± 2.2 | 9.1 ± 2.6 | 0.585 |
| β (95% CI) | 0 (ref) | -0.0 (-0.5 to 0.4) | 0.0 (-0.4 to 0.5) | -0.1 (-0.6 to 0.3) | 0.713 |
| **No** |  |  |  |  |  |
| Mean ± standard deviation | 10.5 ± 2.6 | 10.4 ± 2.8 | 9.2 ± 2.5 | 10.0 ± 2.5 | 0.042 |
| β (95% CI) | 0 (ref) | 0.3 (-0.7 to 1.3) | -0.5 (-1.5 to 0.5) | 0.2 (-0.8 to 1.2) | 0.906 |
| **Diastolic blood pressure** |  |  |  |  |  |
| **Yes** |  |  |  |  |  |
| Mean ± standard deviation | 7.9 ± 2.0 | 7.9 ± 1.9 | 8.0 ± 2.1 | 7.8 ± 2.0 | 0.722 |
| β (95% CI) | 0 (ref) | -0.0 (-0.5 to 0.4) | 0.0 (-0.4 to 0.5) | -0.1 (-0.5 to 0.4) | 0.751 |
| **No** |  |  |  |  |  |
| Mean ± standard deviation | 8.9 ± 2.2 | 8.7 ± 2.6 | 8.1 ± 2.1 | 8.4 ± 1.9 | 0.100 |
| β (95% CI) | 0 (ref) | -0.5 (-1.4 to 0.3) | -0.7 (-1.5 to 0.2) | -0.5 (-1.4 to 0.4) | 0.252 |

Forced expiratory volume in 1 second quartile cut points (lowest to highest quartile):

Men: < 80.7, 80.7 to 91.8, 91.8 to 101.1, and ≥ 101.1.

Women: < 83.8, 83.8 to 95.4, 95.4 to 106.8, and ≥ 106.8.

CI: confidence interval.

Adjustment for demographics (age and sex), behaviors (pack years of cigarette smoking, physical activity, body mass index), co-morbid conditions (diabetes, total and HDL-cholesterol and statin use, history of stroke and history of myocardial infarction), kidney function (estimated glomerular filtration rate and albuminuria), markers of inflammation (C-reactive protein), mean 24-h SBP or DBP and antihypertensive medication classes being taken.
