# Supplementary material for: The association of reduced lung function with blood pressure variability in African Americans: data from the Jackson Heart Study
Source: BMC Cardiovasc Disord. 2016 Jan 12;16:6. doi: 10.1186/s12872-015-0182-2 (PMC4709870; doi:10.1186/s12872-015-0182-2)
Supplement: Additional file 3: Table S3. — Differences in day-night standard deviation of blood pressure across quartiles of forced-vital-capacity by subgroups. (DOCX 28 kb) [file 12872_2015_182_MOESM3_ESM.docx]

Supplemental Table 3. Differences in day-night standard deviation of blood pressure across quartiles of forced-vital-capacity by subgroups.

|  | **Force vital capacity** | | | | |  |
| --- | --- | --- | --- | --- | --- | --- |
|  | Quartile 1  (lowest) | Quartile 2 | Quartile 3 | Quartile 4  (highest) | | p-trend |
|  | **Sex** | | | | |  |
| **Systolic blood pressure** |  |  |  | |  |  |
| **Men** |  |  |  |  | |  |
| Mean ± standard deviation | 9.7 ± 2.3 | 9.3 ± 2.2 | 8.6 ± 2.3 | 9.4 ± 2.3 | | 0.118 |
| β (95% CI) | 0 (ref) | -0.4 (-1.2 to 0.3) | -0.7 (-1.5 to 0.1) | -0.2 (-0.9 to 0.6) | | 0.682 |
| **Women** |  |  |  |  | |  |
| Mean ± standard deviation | 9.6 ± 2.5 | 9.3 ± 2.7 | 9.3 ± 2.5 | 9.2 ± 2.5 | | 0.194 |
| β (95% CI) | 0 (ref) | -0.1 (-0.6 to 0.4) | -0.2 (-0.7 to 0.4) | -0.2 (-0.7 to 0.4) | | 0.501 |
| **Diastolic blood pressure** |  |  |  |  | |  |
| **Men** |  |  |  |  | |  |
| Mean ± standard deviation | 8.2 ± 1.9 | 8.3 ± 2.4 | 7.8 ± 1.8 | 8.2 ± 2.0 | | 0.776 |
| β (95% CI) | 0 (ref) | -0.1 (-0.9 to 0.6) | -0.4 (-1.2 to 0.3) | 0.1 (-0.7 to 0.8) | | 0.976 |
| **Women** |  |  |  |  | |  |
| Mean ± standard deviation | 8.3 ± 2.1 | 8.1 ± 2.2 | 7.9 ± 2.3 | 8.0 ± 2.2 | | 0.133 |
| β (95% CI) | 0 (ref) | -0.4 (-0.9 to 0.1) | -0.6 (-1.0 to -0.1) | -0.3 (-0.8 to 0.2) | | 0.198 |
|  |  |  |  |  | |  |
|  | **Smoking status** | | | | |  |
| **Systolic blood pressure** |  |  |  | |  |  |
| **Never** |  |  |  |  | |  |
| Mean ± standard deviation | 9.5 ± 2.5 | 9.2 ± 2.6 | 9.0 ± 2.4 | 9.2 ± 2.6 | | 0.235 |
| β (95% CI) | 0 (ref) | -0.1 (-0.6 to 0.4) | -0.3 (-0.8 to 0.2) | -0.0 (-0.6 to 0.5) | | 0.724 |
| **Former** |  |  |  |  | |  |
| Mean ± standard deviation | 9.6 ± 2.0 | 9.3 ± 2.4 | 9.1 ± 2.7 | 9.3 ± 2.0 | | 0.545 |
| β (95% CI) | 0 (ref) | -0.0 (-1.0 to 0.9) | -0.1 (-1.1 to 1.0) | -0.3 (-1.3 to 0.6) | | 0.466 |
| **Current** |  |  |  |  | |  |
| Mean ± standard deviation | 10.5 ± 2.9 | 9.9 ± 2.4 | 9.2 ± 2.2 | 9.3 ± 1.8 | | 0.051 |
| β (95% CI) | 0 (ref) | -0.5 (-1.8 to 0.9) | 0.1 (-1.6 to 1.8) | 1.0 (-0.9 to 2.8) | | 0.330 |
| **Diastolic blood pressure** |  |  |  |  | |  |
| **Never** |  |  |  |  | |  |
| Mean ± standard deviation | 8.1 ± 2.0 | 8.0 ± 2.2 | 7.7 ± 1.8 | 8.1 ± 2.1 | | 0.426 |
| β (95% CI) | 0 (ref) | -0.3 (-0.8 to 0.2) | -0.5 (-1.0 to -0.0) | -0.0 (-0.5 to 0.5) | | 0.732 |
| **Former** |  |  |  |  | |  |
| Mean ± standard deviation | 8.2 ± 1.9 | 8.0 ± 2.4 | 8.3 ± 3.1 | 8.1 ± 2.1 | | 0.897 |
| β (95% CI) | 0 (ref) | -0.3 (-1.4 to 0.7) | -0.3 (-1.4 to 0.8) | -0.4 (-1.4 to 0.7) | | 0.536 |
| **Current** |  |  |  |  | |  |
| Mean ± standard deviation | 9.0 ± 2.3 | 8.9 ± 2.0 | 7.8 ± 1.9 | 8.1 ± 2.1 | | 0.042 |
| β (95% CI) | 0 (ref) | -0.6 (-1.8 to 0.7) | -0.7 (-2.2 to 0.8) | -0.1 (-1.8 to 1.5) | | 0.687 |
|  | **Antihypertensive medication use** | | | | |  |
| **Systolic blood pressure** |  |  |  | |  |  |
| **Yes** |  |  |  |  | |  |
| Mean ± standard deviation | 9.8 ± 2.4 | 9.5 ± 2.7 | 9.6 ± 2.6 | 9.7 ± 2.6 | | 0.754 |
| β (95% CI) | 0 (ref) | -0.1 (-0.7 to 0.4) | -0.0 (-0.7 to 0.6) | 0.2 (-0.5 to 0.8) | | 0.578 |
| **No** |  |  |  |  | |  |
| Mean ± standard deviation | 9.2 ± 2.5 | 9.0 ± 2.2 | 8.3 ± 2.0 | 8.7 ± 2.1 | | 0.038 |
| β (95% CI) | 0 (ref) | -0.2 (-0.8 to 0.4) | -0.5 (-1.1 to 0.1) | -0.4 (-1.0 to 0.2) | | 0.118 |
| **Diastolic blood pressure** |  |  |  |  | |  |
| **Yes** |  |  |  |  | |  |
| Mean ± standard deviation | 8.3 ± 2.1 | 8.1 ± 2.6 | 7.9 ± 2.3 | 8.3 ± 2.2 | | 0.974 |
| β (95% CI) | 0 (ref) | -0.3 (-0.8 to 0.3) | -0.5 (-1.1 to 0.0) | 0.1 (-0.4 to 0.7) | | 0.997 |
| **No** |  |  |  |  | |  |
| Mean ± standard deviation | 8.3 ± 2.0 | 8.1 ± 1.7 | 7.7 ± 1.9 | 7.8 ± 2.0 | | 0.024 |
| β (95% CI) | 0 (ref) | -0.4 (-1.0 to 0.2) | -0.5 (-1.1 to 0.1) | -0.5 (-1.0 to 0.1) | | 0.147 |
|  |  |  |  |  | |  |
|  | **Controlled blood pressure status** | | | | |  |
| **Systolic blood pressure** |  |  |  | |  |  |
| **Yes** |  |  |  |  | |  |
| Mean ± standard deviation | 9.3 ± 2.2 | 9.1 ± 2.5 | 8.7 ± 2.3 | 9.2 ± 2.4 | | 0.349 |
| β (95% CI) | 0 (ref) | -0.0 (-0.5 to 0.4) | -0.4 (-0.9 to 0.1) | 0.0 (-0.4 to 0.5) | | 0.767 |
| **No** |  |  |  |  | |  |
| Mean ± standard deviation | 10.6 ± 3.0 | 9.9 ± 2.5 | 10.0 ± 2.6 | 9.6 ± 2.4 | | 0.053 |
| β (95% CI) | 0 (ref) | -0.3 (-1.2 to 0.7) | 0.3 (-0.7 to 1.3) | -0.5 (-1.6 to 0.5) | | 0.563 |
| **Diastolic blood pressure** |  |  |  |  | |  |
| **Yes** |  |  |  |  | |  |
| Mean ± standard deviation | 8.1 ± 2.0 | 7.9 ± 2.1 | 7.5 ± 1.8 | 8.1 ± 2.1 | | 0.609 |
| β (95% CI) | 0 (ref) | -0.3 (-0.8 to 0.1) | -0.7 (-1.1 to -0.2) | 0.0 (-0.4 to 0.5) | | 0.780 |
| **No** |  |  |  |  | |  |
| Mean ± standard deviation | 8.8 ± 2.3 | 8.8 ± 2.6 | 8.5 ± 2.0 | 8.0 ± 2.0 | | 0.036 |
| β (95% CI) | 0 (ref) | -0.1 (-0.9 to 0.7) | 0.3 (-0.6 to 1.1) | -0.7 (-1.6 to 0.2) | | 0.239 |

Forced vital capacity quartile cut points (lowest to highest quartile):

Men: < 81.4, 81.4 to 90.4, 90.4 to 99.8, and ≥ 99.8.

Women: < 83.3, 83.3 to 94.2, 94.2 to 105.2, and ≥ 105.2.

CI: confidence interval.

Adjustment for demographics (age and sex), behaviors (pack years of cigarette smoking, physical activity, body mass index), co-morbid conditions (diabetes, total and HDL-cholesterol and statin use, history of stroke and history of myocardial infarction), kidney function (estimated glomerular filtration rate and albuminuria), markers of inflammation (C-reactive protein), mean 24-h SBP or DBP and antihypertensive medication classes being taken.
