# Supplementary material for: The association of reduced lung function with blood pressure variability in African Americans: data from the Jackson Heart Study
Source: BMC Cardiovasc Disord. 2016 Jan 12;16:6. doi: 10.1186/s12872-015-0182-2 (PMC4709870; doi:10.1186/s12872-015-0182-2)
Supplement: Additional file 4: Table S4. — Difference in day-night standard deviation of blood pressure across quartiles forced-expiratory-volume-in-1-second-to-forced-vital-capacity by subgroups. (DOCX 28 kb) [file 12872_2015_182_MOESM4_ESM.docx]

Supplemental Table 4. Difference in day-night standard deviation of blood pressure across quartiles forced-expiratory-volume-in-1-second-to-forced-vital-capacity by subgroups.

|  | **Forced expiratory volume in 1 second to forced vital capacity ratio** | | | |  |
| --- | --- | --- | --- | --- | --- |
|  | Quartile 1  (lowest) | Quartile 2 | Quartile 3 | Quartile 4  (highest) | p-trend |
|  | **Sex** | | | |  |
| **Systolic blood pressure** |  |  |  |  |  |
| **Men** |  |  |  |  |  |
| Mean ± standard deviation | 9.2 ± 2.2 | 9.2 ± 2.3 | 9.3 ± 2.4 | 9.3 ± 2.2 | 0.616 |
| β (95% CI) | 0 (ref) | 0.4 (-0.3 to 1.2) | 0.2 (-0.6 to 0.9) | 0.2 (-0.5 to 1.0) | 0.714 |
| **Women** |  |  |  |  |  |
| Mean ± standard deviation | 9.4 ± 2.8 | 9.2 ± 2.2 | 9.5 ± 2.5 | 9.3 ± 2.6 | 0.862 |
| β (95% CI) | 0 (ref) | -0.3 (-0.8 to 0.3) | 0.0 (-0.5 to 0.5) | -0.2 (-0.7 to 0.3) | 0.732 |
| **Diastolic blood pressure** |  |  |  |  |  |
| **Men** |  |  |  |  |  |
| Mean ± standard deviation | 8.1 ± 2.2 | 8.3 ± 2.0 | 8.2 ± 1.9 | 7.8 ± 2.0 | 0.308 |
| β (95% CI) | 0 (ref) | 0.4 (-0.3 to 1.1) | 0.0 (-0.7 to 0.8) | -0.4 (-1.1 to 0.4) | 0.218 |
| **Women** |  |  |  |  |  |
| Mean ± standard deviation | 8.1 ± 2.2 | 8.0 ± 2.0 | 8.2 ± 2.5 | 8.1 ± 2.1 | 0.869 |
| β (95% CI) | 0 (ref) | -0.3 (-0.7 to 0.2) | -0.3 (-0.8 to 0.2) | -0.2 (-0.7 to 0.2) | 0.340 |
|  | **Smoking status** | | | |  |
| **Systolic blood pressure** |  |  |  |  |  |
| **Never** |  |  |  |  |  |
| Mean ± standard deviation | 9.3 ± 2.7 | 9.1 ± 2.3 | 9.5 ± 2.6 | 9.1 ± 2.4 | 0.866 |
| β (95% CI) | 0 (ref) | 0.1 (-0.5 to 0.6) | 0.2 (-0.3 to 0.7) | -0.2 (-0.8 to 0.3) | 0.450 |
| **Former** |  |  |  |  |  |
| Mean ± standard deviation | 9.3 ± 2.3 | 9.3 ± 2.2 | 9.0 ± 2.2 | 9.7 ± 2.4 | 0.603 |
| β (95% CI) | 0 (ref) | 0.5 (-0.4 to 1.4) | 0.2 (-0.7 to 1.1) | 0.7 (-0.2 to 1.7) | 0.240 |
| **Current** |  |  |  |  |  |
| Mean ± standard deviation | 9.8 ± 2.4 | 9.5 ± 1.9 | 10.4 ± 2.7 | 10.1 ± 3.0 | 0.472 |
| β (95% CI) | 0 (ref) | -1.0 (-2.6 to 0.5) | -0.0 (-1.7 to 1.6) | 0.7 (-0.8 to 2.1) | 0.218 |
| **Diastolic blood pressure** |  |  |  |  |  |
| **Never** |  |  |  |  |  |
| Mean ± standard deviation | 8.0 ± 2.2 | 8.1 ± 2.1 | 8.0 ± 1.9 | 7.7 ± 2.0 | 0.198 |
| β (95% CI) | 0 (ref) | 0.1 (-0.4 to 0.6) | -0.2 (-0.7 to 0.3) | -0.4 (-0.9 to 0.1) | 0.047 |
| **Former** |  |  |  |  |  |
| Mean ± standard deviation | 8.1 ± 2.3 | 8.0 ± 1.9 | 8.2 ± 3.1 | 8.4 ± 2.2 | 0.602 |
| β (95% CI) | 0 (ref) | 0.2 (-0.8 to 1.2) | 0.2 (-0.8 to 1.2) | 0.2 (-0.9 to 1.2) | 0.735 |
| **Current** |  |  |  |  |  |
| Mean ± standard deviation | 8.5 ± 2.2 | 8.2 ± 1.7 | 9.3 ± 2.5 | 8.6 ± 2.1 | 0.509 |
| β (95% CI) | 0 (ref) | -1.5 (-3.0 to 0.0) | -0.5 (-2.0 to 1.0) | -0.7 (-2.0 to 0.7) | 0.614 |
|  | **Antihypertensive medication use** | | | |  |
| **Systolic blood pressure** |  |  |  |  |  |
| **Yes** |  |  |  |  |  |
| Mean ± standard deviation | 9.7 ± 2.8 | 9.7 ± 2.3 | 9.8 ± 2.6 | 9.6 ± 2.6 | 0.764 |
| β (95% CI) | 0 (ref) | -0.1 (-0.7 to 0.5) | -0.1 (-0.7 to 0.5) | -0.2 (-0.8 to 0.4) | 0.567 |
| **No** |  |  |  |  |  |
| Mean ± standard deviation | 8.8 ± 2.1 | 8.4 ± 2.0 | 9.0 ± 2.4 | 8.9 ± 2.3 | 0.288 |
| β (95% CI) | 0 (ref) | -0.1 (-0.7 to 0.5) | 0.3 (-0.3 to 0.9) | 0.0 (-0.6 to 0.6) | 0.590 |
| **Diastolic blood pressure** |  |  |  |  |  |
| **Yes** |  |  |  |  |  |
| Mean ± standard deviation | 8.3 ± 2.4 | 8.2 ± 2.2 | 8.3 ± 2.4 | 8.0 ± 2.2 | 0.306 |
| β (95% CI) | 0 (ref) | -0.2 (-0.7 to 0.4) | -0.3 (-0.9 to 0.2) | -0.5 (-1.1 to 0.0) | 0.049 |
| **No** |  |  |  |  |  |
| Mean ± standard deviation | 7.8 ± 1.9 | 8.0 ± 1.8 | 8.0 ± 2.0 | 8.0 ± 2.0 | 0.447 |
| β (95% CI) | 0 (ref) | 0.1 (-0.5 to 0.7) | 0.0 (-0.5 to 0.6) | 0.0 (-0.6 to 0.6) | 0.997 |
|  | **Controlled blood pressure status** | | | |  |
| **Systolic blood pressure** |  |  |  |  |  |
| **Yes** |  |  |  |  |  |
| Mean ± standard deviation | 9.0 ± 2.6 | 8.9 ± 2.1 | 9.2 ± 2.3 | 9.1 ± 2.3 | 0.611 |
| β (95% CI) | 0 (ref) | -0.1 (-0.5 to 0.4) | -0.0 (-0.5 to 0.4) | -0.2 (-0.7 to 0.2) | 0.368 |
| **No** |  |  |  |  |  |
| Mean ± standard deviation | 10.1 ± 2.5 | 9.9 ± 2.5 | 10.0 ± 2.8 | 10.1 ± 2.7 | 0.805 |
| β (95% CI) | 0 (ref) | 0.2 (-0.8 to 1.1) | 0.0 (-1.0 to 1.1) | 0.2 (-0.8 to 1.3) | 0.721 |
| **Diastolic blood pressure** |  |  |  |  |  |
| **Yes** |  |  |  |  |  |
| Mean ± standard deviation | 8.0 ± 2.1 | 8.0 ± 1.9 | 7.9 ± 2.0 | 7.8 ± 2.0 | 0.302 |
| β (95% CI) | 0 (ref) | 0.0 (-0.4 to 0.5) | -0.2 (-0.6 to 0.3) | -0.3 (-0.7 to 0.2) | 0.164 |
| **No** |  |  |  |  |  |
| Mean ± standard deviation | 8.6 ± 2.5 | 8.3 ± 2.2 | 8.6 ± 2.2 | 8.7 ± 2.1 | 0.538 |
| β (95% CI) | 0 (ref) | -0.3 (-1.2 to 0.5) | -0.3 (-1.2 to 0.6) | -0.1 (-1.0 to 0.8) | 0.796 |

Forced expiratory volume in 1 second: Forced vital capacity ratio quartile cut points (lowest to highest quartile):

Men: < 0.96, 0.96 to 1.02, 1.02 to 1.07, and ≥ 1.07.

Women: < 0.97, 0.97 to 1.03, 1.03 to 1.07, and ≥ 1.07.

CI: confidence interval.

Adjustment for demographics (age and sex), behaviors (pack years of cigarette smoking, physical activity, body mass index), co-morbid conditions (diabetes, total and HDL-cholesterol and statin use, history of stroke and history of myocardial infarction), kidney function (estimated glomerular filtration rate and albuminuria), markers of inflammation (C-reactive protein), mean 24-h SBP or DBP and antihypertensive medication classes being taken.
